# Identification of novel cellular clusters define a specialized area in the cerebellar periventricular zone

**DOI:** 10.1038/srep40768

**Published:** 2017-01-20

**Authors:** María Alejandra González-González, Gabriela B. Gómez-González, Marymar Becerra-González, Ataúlfo Martínez-Torres

**Affiliations:** 1 Instituto de Neurobiología, Universidad Nacional Autónoma de México, Departamento de Neurobiología Celular y Molecular, Laboratorio de Neurobiología Molecular y Celular, Juriquilla, Querétaro, 76230, México

## Abstract

The periventricular zone of cerebellum is a germinative niche during the embryonic development, nevertheless its structural organization and functional implications in adult have not been widely studied. Here we disclose the presence of two novel clusters of cells in that area. The first one was named the subventricular cellular cluster (SVCC) and is composed of cells that express glial and neuronal markers. The second was named the ventromedial cord (VMC) and appears as a streak of biciliated cells with microvillosities facing the ventricle, that includes GFAP^+^ and nestin^+^ cells organized along the periventricular vasculature. The dorsal limit of the SVCC is associated with myelinated axons of neurons of unknown origin. This paper describes the characteristics and organization of these groups of cells. They can be observed from late embryonic development in the transgenic mouse line GFAP-GFP. The SVCC and VMC expand during early postnatal development but are restricted to the central area of the ventricle in adulthood. We did not find evidence of cell proliferation, cell migration or the presence of fenestrated blood vessels. These findings provide new insights into the knowledge of the cellular composition and structural organization of the periventricular zone of cerebellum.

It has been shown previously that the subventricular zone of the cerebellum exhibits a diversity of cells that display different electrophysiological characteristics^1^. Whole-cell patch-clamp revealed that electric responses obtained from the subventricular zone of the cerebellum, which corresponds to lobes I and X, include neurons, astrocytes, oligodendrocytes and stem-cell like cells^1^. This evidence is consistent with other observations that demonstrated the presence of ependymal ciliated glial cells and fibers that seem to correspond to axons^2^. Hitherto, as far as we know, structural evidence for the presence of oligodendrocytes or an active niche of stem cells on the roof of the fourth ventricle has not been shown. In our previous report^1^, we provided evidence that cells forming the ependymal glial cell layer of the roof of the fourth ventricle respond to γ-aminobutyric acid (GABA), a neurotransmitter known for its modulatory role in neurogenesis and cell migration in the lateral ventricles^3^,^4^,^5^,^6^.

Whereas the role of GABA neurotransmission in the periventricular area of the lateral ventricles has received much attention due to the presence of an active neurogenic niche^7^,^8^, information concerning the cellular diversity and organization of the ependymal surface of the roof of the fourth ventricle is relatively scarce^2^,^9^. We do not yet know the source of GABA that may evoke the electric responses generated by ependymal glial cells; however, the participation of the GABA-A receptors was established based on their electrophysiological and pharmacological characteristics: chloride currents blocked by bicucculline and 1,2,5,6-Tetrahydropyridin-4-yl methylphosphinic acid (TPMPA), as well as assessed by *in situ* hybridization and immunofluorescence^1^. It is well known that GABA, acting through GABA-A receptors, triggers the differentiation of neuroblasts in postnatal subventricular zones. Thus, we considered worthwhile to explore the organization of the periventriclular zone of the cerebellum in which we reported the presence and electrophysiological profile of GABA-A receptors.

A visual inspection of a 1-mm toluidine blue-stained coronal section of the roof of the fourth ventricle (lobes I and X) revealed that the area from which the electrophysiological GABA responses were obtained^1^ forms an isolated cluster of cells with heterogeneous characteristics (Fig. 1A–C). This cluster is limited dorsally by the terminal feet of the Bergmann glia and ventrally by the ependymal glial cell layer (Fig. 1J, yellow and green arrowheads, respectively). We will refer to this structure as the subventricular cellular cluster (SVCC). A possible explanation for the presence of the SVCC is that this structure corresponds to remnants of proliferative zones that are active during early development or that it is a specialized zone integrating signals among the parenchyma, cerebrospinal fluid and blood. To examine these possibilities and to explore potential roles for this cluster of cells we used a combination of techniques that include immunofluorescence, CLARITY, transmission and scanning electron microscopy as well as electrophysiology. Finally, we also tested the proliferation potential of the SVCC cells.

## Materials and Methods

### Ethics statement

All protocols and procedures were approved by the Bioethics Committee of the Instituto de Neurobiología, Universidad Nacional Autónoma de México (INB-UNAM license: INEU/SA/CB089) in accordance with the rules and regulations of the Society for Neuroscience: Policies on the Use of Animals and Humans in Neuroscience Research and on local and international bioethical guidelines including the NOM-062-ZOO which is in accordance with the recommendations of the National Institutes of Health publication: “Guide for the Care and Use of Laboratory Animals”.

### Animals

CD1 and transgenic GFAP-GFP mice^10^ from embryonic (E) to postnatal day (P) 30 to P60 were obtained from the local vivarium.

### Immunohistofluorescence

P30 male CD1 mice were deeply anesthetized with pentobarbital (30 mg/kg) and intracardially perfused with saline solution followed by PFA (4%) in phosphate-buffered saline (PBS), pH 7.2. For whole mount immunolabeling, PFA (4%) in PBS plus Triton (0.1%) was used^11^. For slices, cerebella were processed as previously reported^12^. The following antibodies were used in a 1:200 dilution: nestin (Santa Cruz, sc-21249), vimentin (Sigma, V4630), calbindin (Santa Cruz, sc7691), calretinin (Santa Cruz, sc11644), myelin basic protein (Abcam, ab40390), NG2 (Santa Cruz, sc30923) Ki67 (Abcam, ab15580), GAD65 (Santa Cruz, sc32270), NeuN (Millipore, MAB377), doublecortin (Sigma, D9693) and BrdU (Santa Cruz, 3H579). Secondary antibodies coupled to Alexa Fluor 488 or 594 (Invitrogen) were used (1:200 dilution). Propidium iodide (0.05 mg/mL) with RNAse (0.01 μg/mL) or 4′,6-diamidino-2-phenylindole (DAPI, 0.01 mg/mL) were used to stain cell nuclei. Preparations were processed for imaging in a confocal microscope (Zeiss, LSM-780), 15- to 20-μm z-stacks for coronal slices and 200- to 300-μm z-stacks for whole mount preparations were obtained with 10 X and/or 25 X objectives (n = 4 for each antibody tested).

The postnatal array of glial cells in the SVCC and the surface of the roof of the fourth ventricle were analyzed in transgenic GFAP-GFP male mice at postnatal ages: P0, P5, P10, P15, P20 and P30 (n = 3, each age). The brains were processed as described in the section on immunofluorescence; 40-μm slices that included lobules I and X were collected and cell nuclei stained with propidium iodide (0.05 mg/mL) including RNAse (0.01 μg/mL), followed by dehydration in 96% and 100% ethanol and finally mounted in Vectashield (Vector Laboratories). Focal sections of 0.88 μm were obtained from 40-μm slices in a Zeiss LSM 510 confocal microscope.

### Assays of cell proliferation

Two groups of P30 male CD1 mice were assessed; the first group was tested for rapid cell proliferation (four hours) and the second one for slow cell proliferation (five days). For the first group, either intraperitoneal BrdU (50 mg/kg) or PBS (n = 5) was administrated, and four hours later the mice were anesthetized by intraperitoneal administration of pentobarbital (30 mg/kg) and intracardially perfused as indicated in the immunofluorescence section. To test for slow proliferation two doses of BrdU (50 mg/kg) were administrated to the second group (n = 5) every six hours over four consecutive days, and on the fifth day mice were intracardially perfused with PFA (4%); brains were postfixed overnight at 4 °C and cryoprotected with three sucrose gradients (10–30%). Forty-μm slices were obtained and treated with HCl 2 N for 1 hour at 37 °C. An antibody against BrdU (Santa Cruz, 3H579) was used and detected with a secondary antibody coupled to Alexa 488 (Invitrogen, A-11006), and cell nuclei were stained with DAPI. Samples were observed in a Zeiss LSM 780 confocal microscope, and 1.0-μm optical slices were obtained from 40-μm stacks.

### Test for fenestrated capillaries

To evaluate whether capillaries from the SVCC are fenestrated, Evans blue solution (4% in physiologic saline solution) was administrated intraperitoneally (100 mg/kg) in 5 adult mice. When the skin of the mice turned blue (after 24 h), the mice were anesthetized and intracardially perfused with physiological saline solution. Brains were dissected immediately, and 50-μm slices were obtained using a vibratome (Leica, VT1000S). In addition, slices were labeled with the fluorescent marker for Nissl substance (Neuro Trace, Nissl stain, Molecular Probes, N-21480) and mounted. Samples were observed under a Zeiss LSM 780 confocal microscope, and 1.0-μm optical slices were obtained from 40-μm stacks.

### mCherry adenoviral labeling and DiI labeling

We used a recombinant serotype 5 adenovirus (AdV) carrying mCherry under the control of the CMV promoter-enhancer (Vector BioLabs, Philadelphia, USA). The virus was stereotaxically administered (3.3 × 10^7^ viral particles/μl at constant flow for 2 min) with a Hamilton Neuro syringe (Hamilton, 1710) into the parenchyma of the cerebellum (Bregma −6.36 mm, ventral −3.30 mm and lateral 0.0 mm, according to the Mouse Brain Atlas^13^) of anesthetized 2-month-old GFAP-GFP transgenic male mice (10 μL/50 g of ketamine/xylazine 7:3). After 72 h, mice were processed as described in the immunohistochemistry section (n = 6), cerebella were sliced into 45-μm thick coronal sections using a cryostat (Leica, CM3050s), the slices were collected and the nuclei were stained with DAPI (1 mg/mL). Samples were observed under a Zeiss LSM 510 confocal microscope, and 25- to 40-μm stacks of 1.0-μm optical slices where obtained.

For DiI (1,1′-Dioctadecyl-3,3,3′,3′-tetramethylindocarbocyanine perchlorate) staining, fixed brains were processed as described above (n = 3), a needle was impregnated with the tracer and applied 200 μm anterior to the SVCC in the whole cerebellum for 1 h and then the brains were incubated at 25 °C for three months, 40-μm slices were obtained, stained with DAPI and mounted to be analyzed in a Zeiss LSM 780 confocal microscope.

### CLARITY

For understanding the organization of astrocytes in the SVCC during development we performed CLARITY^14^ in whole brains of transgenic GFAP-GFP mice at embryonic ages (E10, E12, E15, E18, E19, n = 4) and postnatal ages (P0, P5, P10, P15, P20, P30, n = 4). Mice were intracardially perfused with PBS, followed with a cold Hydrogel Monomer Solution (HMS) containing: acrylamide (4.0%), bis-acrylamide (0.05%), A-044 initiator (0.25%, Wako VA044), PBS and PFA (4.0%); once the tissue was perfused it was immersed in HMS for 6–10 days at 4 °C, the tissue was then placed into a 15-mL polypropylene centrifuge tube with fresh HMS; polymerization was induced at 37 °C and allowed to proceed for 3 h. The HMS was removed, and brains were sectioned into 2.0-mm thick slices and immersed in a Clearing Solution (CS: 200 mM boric acid, 4% SDS, diluted with H_2_O, pH 8.5). Passive CLARITY was performed at 37 °C in a shaker incubator at 80 rpm. After brain clarification (approximately one month), the tissue was washed twice for 24 h in PBST (0.1% Triton in 1X PBS) at 37 °C, 80 rpm. Cell nuclei were stained with propidium iodide in PBST for 24 h at 37 °C, 80 rpm. Tissue was then mounted in glycerol (80%) and processed in a confocal microscope (Zeiss LSM 510); 2- to 5-μm optical slices were obtained from 380- to 600-μm stacks.

### Scanning and transmission electron microscopy

The ultrastructure of the SVCC and surface of the roof of the fourth ventricle were analyzed by transmission and scanning electron microscopy (TEM and SEM, respectively). TEM was performed as previously reported^12^; 60– to 70-nm ultrathin slices were obtained in an ultramicrotome, mounted on copper grids and observed in a transmission electron microscope (JEOL JEM-1010 model, at 80 KV), n = 3. For SEM, the samples were dissected to expose the surface of the ventricle (see supplementary Fig. 1). Samples were dehydrated in a Tousimis dryer (Samdri-PVT-3D), covered in gold in a sputter coater (Denton Vacuum) and processed in a scanning electron microscopy (Zeiss, EVO 50), n = 3.

### Whole-cell patch-clamp electrophysiology

Coronal slices of cerebellum (250 μm thick) from 50- to 60-day-old C57 male mice that were anesthetized with isofluorane, were collected in an ice cold carbogen-bubbled sucrose-rich solution containing in mM: sucrose (212.7), KCl (5.0), NaH_2_PO_4_ (1.25), MgSO_4_ (3.0), CaCl_2_ (1.0), NaHCO_3_ (26.0), and glucose (10.0), pH 7.4. Slices were incubated for 30 min at room temperature in this solution. Whole-cell patch clamp was performed at 37 °C in artificial cerebrospinal fluid solution (ACSF) containing in mM: NaCl (125.0), KCl (2.5), NaH_2_PO_4_ (1.25), MgCl_2_ (1.0), CaCl_2_ (2.0), NaH_2_PO_4_ (1.25), NaHCO_3_ (26.0) and glucose (10.0), pH 7.4. Electrical stimulation was performed using a 6–8 MΩ patch pipette containing in mM: K-gluconate (130), KCl (7), ATP-Mg (4.0), GTP-Na (0.3), phosphocreatine-di (Tris) (10), HEPES (10) and biocytin (0.4%), pH 7.2, 300 mOsm. Cells were visualized under a Nikon FN-S2N microscope. Whole-cell patch-clamp recordings were performed using a Multiclamp 700B amplifier (Molecular Devices). For characterizing the electrical profiles of the cells from the SVCC we applied three current protocols: (i) cells held at−80 mV, 160-msec voltage steps from −100 to +20 mV in 10-mV increments; (ii) cells held at −20 mV, 160-msec voltage steps from −40 to +150 mV in 10-mV increments; and (iii) cells held at −40 mV with 160-msec voltage steps from −60 to +40 mV in 10-mV increments. As the experiments progressed we established that these protocols disclosed diverse cell populations with different electrical responses.

To reveal the morphology of recorded cells, they were filled with biocytin, the slices were fixed in PFA (4%) for at least 12 h (4 °C), endogenous peroxidase activity was blocked by incubating in hydrogen peroxide (3%) for 30 min, and avidin-biotin complexes were formed using the Vectastain ABC kit and detected with diaminobenzidine (Vector Laboratories). The 250-μm slices were cryoprotected and embedded in Tissue Tek O.C.T. (Sakura) to obtain 40-μm thick slices. Serial images from different focal depths were obtained in a light microscope (Olympus BX60). Image processing was performed with Helicon Focus software. Biocytin-labeled cells were hand drawn on paper using a *camera lucida*.

## Results

### Location and cellular composition of SVCC and VMC

**Figure 1:**
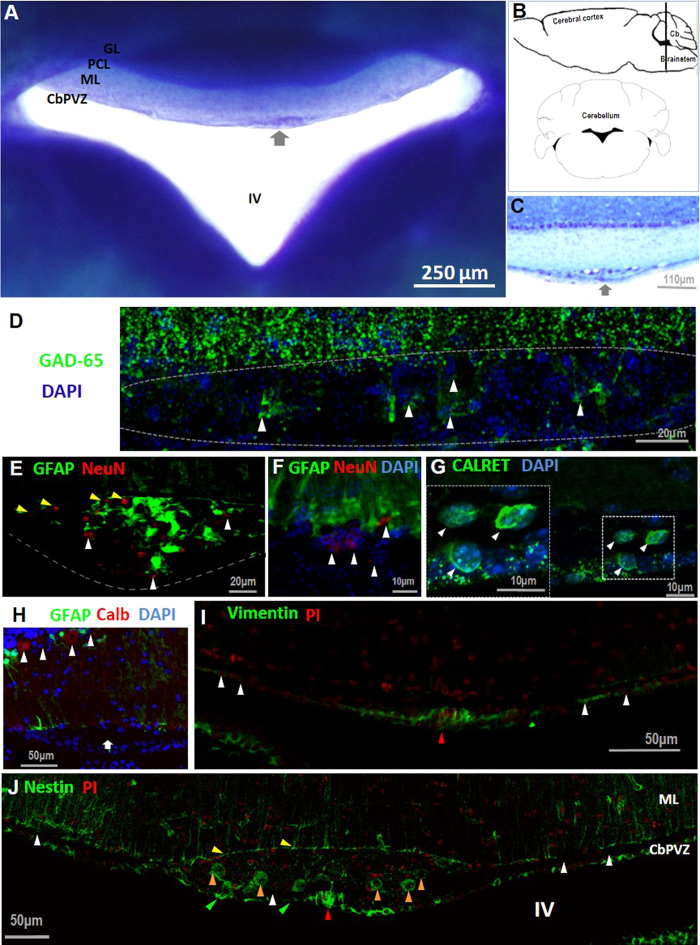
Position and organization of the subventricular cellular cluster. (**A**) and (**C**), toluidine blue stain of 1-mm and 40-μm (respectively) coronal cerebellar slices; an arrow indicates the location of the SVCC in the CbPVZ. (**B**) a black line indicates the location of the SVCC in a sagittal plane, and below (**C**), it is shown in the coronal plane. (**D**). White arrowheads point to cells positive for GAD-65 in the SVCC. (**E**) Cells expressing the neuronal NeuN marker are distributed in the SVCC (white arrowheads); some of the cells are in vicinity with the terminal end feet of Bergmann glia (yellow arrowheads in E and white arrowheads in F). (**G**) Calretinin-positive cells are distributed throughout the SVCC (white arrowheads); the insert shows an amplification. (**H**) The SVCC does not include calbindin-positive cells (arrow), in contrast to Purkinje neurons (arrowheads). (**I**) Vimentin label was found in a population of ependymal glial cells (red arrowhead) but absent in the SVCC. (**J**) SVCC includes cells positive for nestin (orange arrowheads), and is delimited dorsally by Bergmann glia (yellow arrowheads) and ventrally by ependymal cells (green arrowheads). Cb: cerebellum; GL: granular layer; PCL: Purkinje cellular layer; ML: molecular layer; CbPVZ: cerebellar periventricular zone; IV: fourth ventricle; PI: propidium iodide; CALRET: calretinin; GFAP: glial fibrillar acidic protein; Calb: calbindin; GAD-65: glutamate decarboxylase isoform 65.

Serial coronal sections of cerebellum were stained with toluidine blue as shown in Fig. 1A. A 1.0-mm slice evidenced the SVCC towards the central area of the roof of the ventricle (Fig. 1A, arrow), and consecutive 40-μm slices from 5 male mice indicated that the dimensions of the SVCC are 300 ± 20 μm on the lateral extension and 160 ± 20 μm along the anterior-posterior axis (arrow in Fig. 1C). The SVCC was composed on average of 314 ± 8 cells of diverse morphology.

Our previous report described electrophysiological responses that correspond to neurons and other cells that responded to GABA through activation of GABA-A receptors^1^. Thus, to determine whether GABAergic cells were present in the SVCC, we used a selective antibody for GAD-65, the enzyme necessary to synthesize the GABA used in neurotransmission and associated with nerve terminals (white arrowheads in Fig. 1D). From three cerebella examined we found an average of 33 ± 1 GAD-65 positive cells. Further evidence of the presence of neurons in the SVCC was provided by immunolabeling with NeuN antibody, a selective marker of neurons, that indicated that on average 65 ± 4 neurons were present in the SVCC (arrowheads in Fig. 1E,F). In addition, an antibody selective for calretinin, a marker of interneurons in different areas of the brain, labeled the soma of several (10 ± 2) cells (arrowheads in Fig. 1G); an antibody selective for the calcium-binding protein calbindin did not label cells in the area, whereas Purkinje neurons were positive (arrow and arrowheads in Fig. 1H).

Since our electrophysiological recordings suggested the presence of precursor cells in this area^1^ we tested for the expression of the intermediate filaments nestin and vimentin. No vimentin signal was observed in the SVCC, whereas scattered label was detected along the ependymal glial layer (arrows in Fig. 1I). A number of cells (39 ± 7) positive for nestin were labeled in the SVCC (orange arrowheads in 1J); these cells possess large soma and are embedded within the SVCC. A second cellular population of nestin^+^and vimentin^+^cells forms a compact cluster of cells detected medially in the ependymal layer (red arrowhead in Fig. 1I,J, respectively); however, when consecutive serial slices from this area were examined, it was clear that these cells form a stream distributed ventromedially along the surface of the roof of the ventricle, in intimate contact with the cerebrospinal fluid (see below). We will refer to this array of cells as the ventromedial cord (VMC). Along with nestin^+^cells from the VMC, we also found GFAP^+^cells, some of which were positive for both markers (see below).

**Figure 2:**
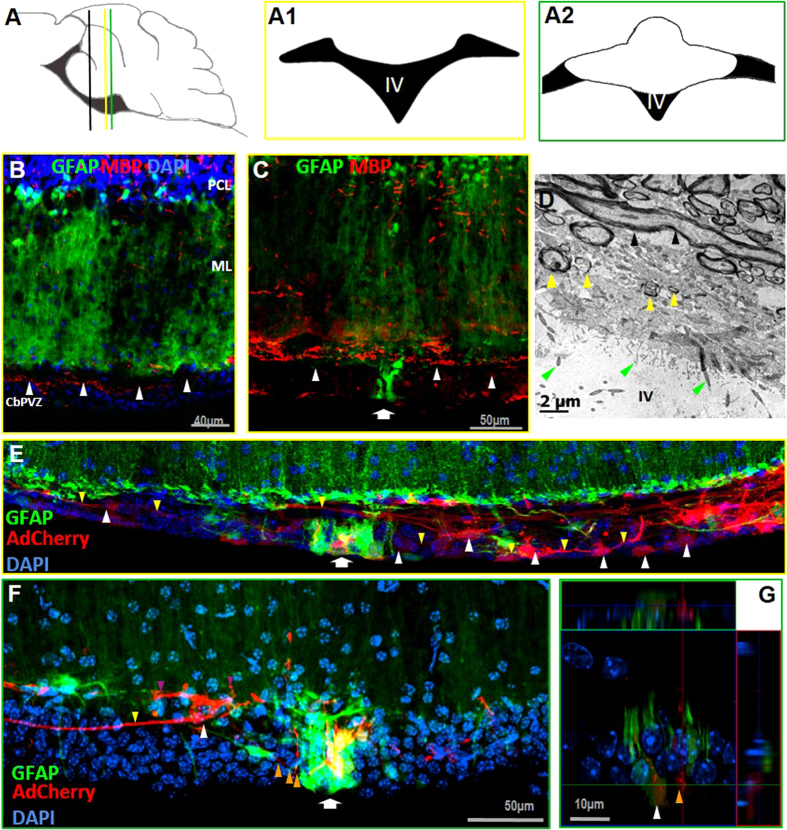
Evidence of neurons in and around the SVCC. (**B**) Cells labeled by MBP are distributed through the SVCC (white arrowheads), and the VMC is disclosed by the expression of GFP (arrow in **C**). (**D**) Transmission electron microscopy showed the presence of transversal and longitudinal axons (black and yellow arrowheads), the presence of multiciliated cells indicated the presence of ependymal cells (green arrowheads). A sagittal representation of the site of AdCherry administration (black line in panel A); yellow and green lines indicate the coronal planes represented in A1 and A2. (**E**) mCherry randomly labeled cells in the SVCC (white arrowheads); several cells present long processes (yellow arrowheads) that extend laterally. The VMC is indicated with an arrow (**E**,**F**), and it includes GFAP^+^(in green) and GFAP^-^ cells, some cells labeled with mCherry (orange arrowhead in **G**) and several cells labeled with both fluorescent proteins. In (**F**) Individual cell labeling was obtained with mCherry (white arrowhead) and identified one cell extending a long projection laterally (yellow arrowhead); in addition, GFAP + cells, morphologically similar to astrocytes, project several processes to the VMC (orange arrowheads) whereas others contact the terminal feet of Bergmann glia (purple arrowheads). In (**G**) an orthogonal plane of 2F is presented. GFAP: glial fibrillar acidic protein; AdCherry: adenoviral vector carrying the fluorescent protein Cherry gene; CbPVZ: cerebellar periventricullar zone.

Electrophysiological evidence suggested the presence of neurons and oligodendrocytes in the SVCC; therefore, we used an antibody selective for myelin basic protein (MBP), which is the main component of the myelin membrane of oligodendrocytes. Distribution of MBP was sparse in cells facing the ventricle and more abundant in cells located towards the dorsal side of the SVCC, at the interface between the Bergmann glia end feet (arrowheads in Fig. 2B,C). The presence of myelinated axons was further confirmed by transmission electron microscopy (Fig. 2D), which showed many processes running along the transversal and longitudinal planes, isolating the cells of the SVCC from the end feet of Bergmann glia. We observed neither active synaptic zones in multiple images captured from this area nor contacts of Bergmann glia with those axons. The origin and destiny of these processes is still unknown.

### Adenoviral labeling

To determine the morphology of individual cells of the SVCC and VMC we injected into the cerebellar parenchyma adenoviruses carrying mCherry, using a low concentration of viruses to achieve scattered, non-selective labeling of different cells. In the SVCC we observed 23 ± 2 cells (n = 5) with a large nucleus (23 ± 3 μm diameter; white arrowheads in Fig. 2E,F) that extended two main processes contralaterally (yellow arrowhead), and form an array of “beads on a string”, from these cells 9 ± 1 cells colocalize with the GFP. In this report we do not provide unequivocal evidence that these cells can be properly classified as neurons, and more studies are necessary to determine their electrical properties and role in this area of the cerebellum.

The mCherry adenovirus randomly labeled cells from the VMC, which defined the organization of this group of cells. The VMC is composed of GFAP^−^ and GFAP^+^cells (yellow and white arrowheads, respectively, in Fig. 2G, Supplementary Fig. 4–5) that have the nucleus towards the ventricular surface and extend their processes toward the parenchyma; two or more astrocytes extend their processes to the VMC, which was closely associated with the microvasculature (orange arrowhead in Fig. 2F).

**Figure 3:**
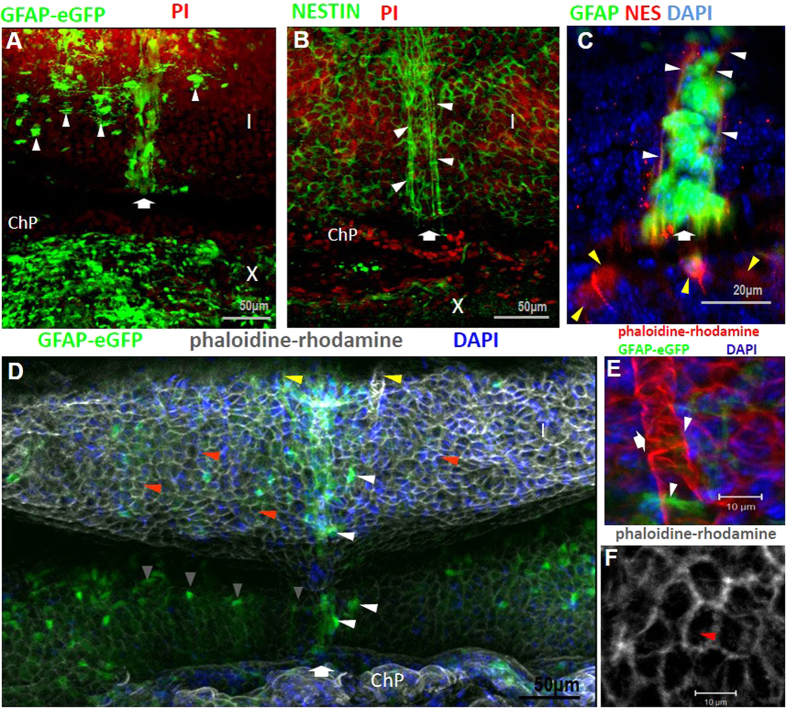
Distribution of ventromedial cord on the roof of the fourth ventricle. The distribution of the VMC on lobe I (I) is indicated with an arrow in (**A**,**B**,**C** and **D**) In A GFAP^+^cells are aligned ventromedially; additionally, several individual glial cells with large somas were found lateral to the VMC (white arrowheads). (**B**) Cells positive for nestin are distributed in the VMC (white arrowheads), and in (**C**) white arrowheads point to some nestin^+^cells in the VMC (labeled in red), and yellow arrowheads indicate nestin^+^cells lateral to the VMC. (**D**) Phaloidine-rhodamine showed the surface of the roof of the fourth ventricle in the GFAP-GFP transgenic mouse. The VMC is indicated by white arrowheads; two capillaries run along this structure (yellow arrowheads in **D** and white arrow in **E**), and they are contacted by long glial processes extending from the VMC (arrowheads in **E**). Some glial cells with radial morphology extend longitudinally along the interface between the choroid plexus (ChP) and lobe I (gray arrowheads in **D**). (**F**) Pinwheel-like structures are present on the surface of the ventricle (red arrowheads in **D** and **F**). PI: propidium iodide; NES: nestin; GFAP: glial fibrillar acidic protein; I: lobe I; ChP: choroid plexus.

To gain some understanding of how the VMC contacts the ventricle, we processed cerebella from transgenic GFAP-GFP mice to expose the surface of the ventricle, then we labeled cells with an antibody against nestin. Towards the central area of the cerebellum a stream of GFAP^+^cells was observed; these cells extend their processes rostrocaudally (arrow in Fig. 3A). This organization contrasts with GFAP^+^cells from the rest of the roof of the ventricle, which show mostly multiciliated ependymal cells. This array was further observed by nestin labeling (arrow in Fig. 3B) that revealed the stream of cells towards the central region (arrowheads) and an array similar to a honeycomb in the rest of the roof of the ventricle. When the antibody that recognizes nestin was used on GFAP-GFP mice, we observed that many cells of the stream are positive to both markers, whereas others remain negative to nestin (yellow and white arrowheads in Fig. 3C). Therefore, in the VMC, GFAP^+^cells intermingle with nestin^+^cells and a large population is GFAP^+^/nestin^+^.

To document the organization of the VMC along the roof of the fourth ventricle and its association with blood vessels, we labeled the cell-cytoskeleton with phalloidin-rhodamine in GFAP-GFP mice (Fig. 3D–F). The stream of GFAP^+^/nestin^+^cells was found along the rostro-caudal axis, in the central region of lobes I and X (arrow in 3 D). GFAP^+^cells of the VMC were in intimate contact with capillaries (diameter 18.8 ± 1.5 μm, mean ± SD) that run in parallel to this structure along the anterior-posterior plane (yellow arrowheads in Fig. 3D). GFAP^+^cells on the surface of the ventricle extend their processes to the microvasculature, contacting endothelial cells (Fig. 3E).

DiI labeling showed that cells of the VMC incorporate into the cerebellar parenchyma between lobes I and X (supplementary Fig. 3), but the destiny of these cells was not determined. Analysis of the ventricle surface labeled with phalloidin-rhodamine (Fig. 3D and F, red arrowheads) revealed scattered cell arrays similar to the pinwheel array associated with neurogenic niches in the lateral ventricles^15^.

**Figure 4:**
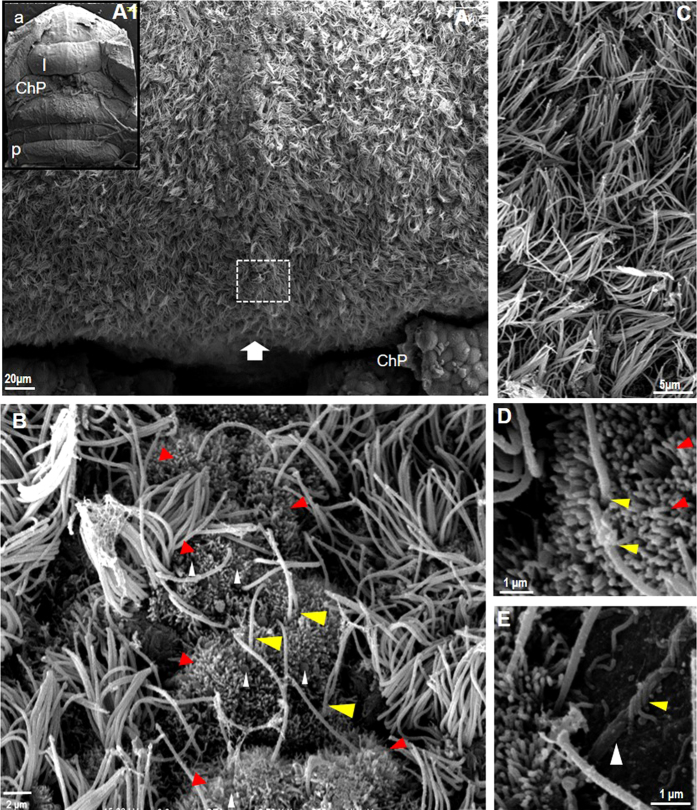
Surface of the roof of the fourth ventricle. SEM image in A1 shows a panoramic view of the ventricle, with lobe I (I) anterior to the choroid plexus (a, anterior, p, posterior). Magnification of the area shows the cilia that characterize the ependymal cells and a thickening that corresponds to the VMC (white arrow); this structure includes cells with only two cilia (yellow arrowheads in **B**,**D**) and with multiple small cilia (red arrowheads in **B**,**D**). These cells also contain several spheroid structures (white arrowheads in **B**), and blood vessels were observed near this area (white arrowhead in **E**). The VMC is different from the uniform cellular organization found laterally that consists of multiple ciliated cells shown in (**C**) I: lobule I; ChP: choroid plexus; a: anterior; p: posterior.

Analysis of this area by SEM revealed a thickening in the central area of the roof of the fourth ventricle that corresponds to the VMC (arrow in Fig. 4A). The ventricular surface of these cells possesses two cilia and multiple microvilli (yellow and red arrowheads respectively in Fig. 4B and D); blood vessels were surrounded and contacted by cell processes, most probably from astrocytes (white and yellow arrowheads in Fig. 4E). In contrast, cells distributed laterally to the VMC present multiple cilia, a common feature of ependymal glial cells of the ventricles (Fig. 4C).

### Assays of cell proliferation and fenestrated capillaries

**Figure 5:**
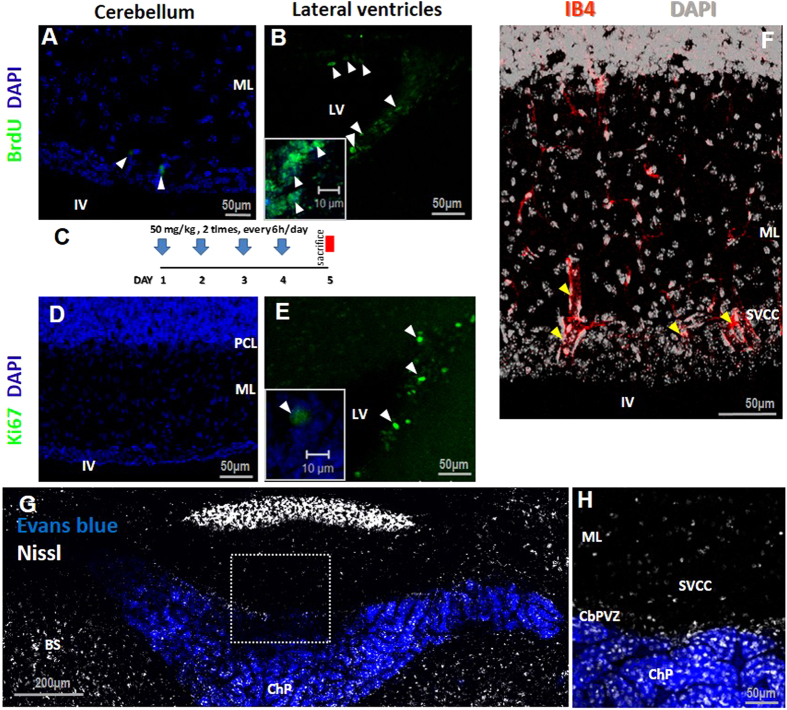
Proliferation assay and blood vessels of the SVCC. (**A**) BrdU labeling assay (administration protocol in **C**). Several cells were identified in the SVCC associated with blood vessels (arrowheads in **A**); no signal for Ki-67 or incorporation of BrdU was detected (**A,D**), whereas cells in the lateral ventricle were positive. (**F**) Blood vessels were observed in the SVCC by IB4 (yellow arrowheads). (**G**,**H**) Evans blue did not label the SVCC, whereas the choroid plexus (ChP) readily incorporated the stain. LV: lateral ventricles; IV: fourth ventricle; ML: molecular layer; SVCC: subventicular cellular cluster; CbPVZ: cerebellum periventricular zone; ChP: choroid plexus; IB4: isolectin IB4.

The presence of GFAP^+^/nestin^+^cells surrounding blood vessels on the ventricular surface led us to test their potential to integrate BrdU and for immunolabeling with Ki67. The ability to incorporate BrdU was compared with the cells from lateral ventricles, where high rates of cell proliferation are well known^16^. Two protocols were used (see methods); the rapid proliferation test did not show evidence of cells that incorporated BrdU. On the other hand, BrdU was integrated in a small, but consistent number of cells of the SVCC in the slow incorporation protocol (6 ± 2 BrdU^+^, n = 5); the BrdU^+^cells were found to be associated with blood vessels (Fig. 5A, white arrowheads). This incorporation contrasted with the high number of BrdU^+^cells in the lateral ventricles (Fig. 5B). Ki67 was not detected in the SVCC (5D), whereas the lateral ventricle exhibited strong labeling (white arrowheads in Fig. 5E). This finding is consistent with the well-known limited rate of neurogenesis in the cerebellum. We then determined whether this area is associated with blood irrigation by using the glycoprotein isolectin IB4 to label endothelial cells of blood vessels. As illustrated in Fig. 5F, capillaries (18.8 ± 1.5 μm diameter) were observed in all extensions of the SVCC (yellow arrowheads), which contrasted with the discrete labeling of IB4 in the capillaries of the lateral areas of the roof of the ventricle (10.2 ± 1.60 μm diameter). We then tested whether these blood capillaries are fenestrated, as are capillaries of circumventricular organs (CVOs), by administrating the albumin-binding dye Evans blue. In seven independent assays we found no evidence of dye incorporation in the SVCC (square in Fig. 5G), whereas the choroid plexus, a CVO with fenestrated capillaries, readily incorporated the dye (ChP in Fig. 5G,H).

### Electrophysiological characteristics of cells in the SVCC

**Figure 6:**
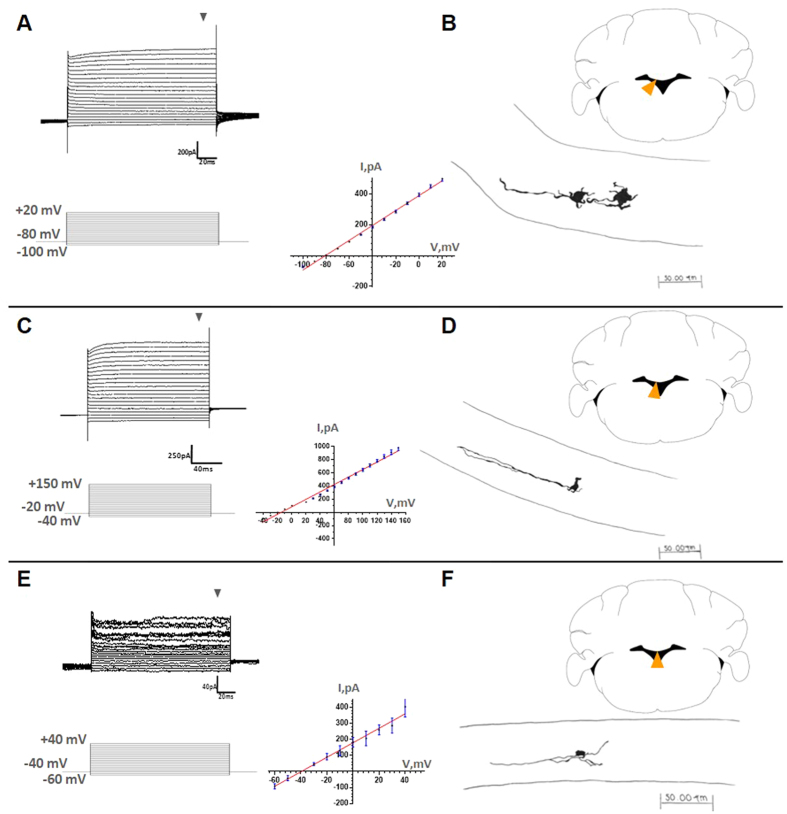
Electrophysiological characteristics of cells of the SVCC. Whole-cell patch-clamp sample recordings from cells at different locations in the SVCC (orange arrowheads in coronal cerebellar slices). Different current profiles were dissected after application of the protocols indicated (**A**–**C** and **E**). Recordings in (**A**–**C**) correspond to glial cells, while E is similar to those of neuroblasts of the lateral ventricles. Notice the linear current-voltage relation (I–V plots); these were calculated at the end of the current steps (gray arrowheads). During the recordings, the cells were filled with biocytin. The current profiles correlate with diverse cell morphologies unveiled by drawing on paper the biocytin labeled cells, using a *camera lucida* (**B**, **D**–**F**). Cells in B were located in the lateral portion of the SVCC and possess large soma (20–25 μm), short projections and one long processes that projects laterally. In D, cells with smaller soma (5–8 μm) and two long lateral processes; in F, cells were distributed medially, with small soma (8-10 μm) and multiple projections extended transversally in the medial portion of the SVCC.

We performed whole-cell path-clamp recordings in acute slices from cerebellum; cells with different morphology were analyzed. We noticed that these cells presented low input resistance and were divided in two populations: 1) cells with resistance of 40–50 MΩ (Fig. 6A–D) and 2) cells with resistance of 18–20 MΩ (Fig. 6E,F). Three different current profiles were observed; the linear current-voltage relations are plotted in Fig. 6A,C and E. The electrophysiological characteristics correlate with the morphology and position of the cells: 1) a cell population located in the lateral portion of the SVCC, with larger soma (20–25 μm), short projections and one long process that projects laterally (Fig. 6B); 2) cells with smaller soma (5–8 μm) and two long lateral processes (Fig. 6D); 3) cells with small soma (8–10 μm) and multiple projections extending transversally into the medial portion of the SVCC (Fig. 6F); these cells also present different current profiles (Fig. 6E), similar to neuroblasts from the subventricular zone of the lateral ventricles^7^, and in contrast with the profiles of the cells in 6B and D that correspond to glial cells.

### Development of SVCC and VMC

**Figure 7:**
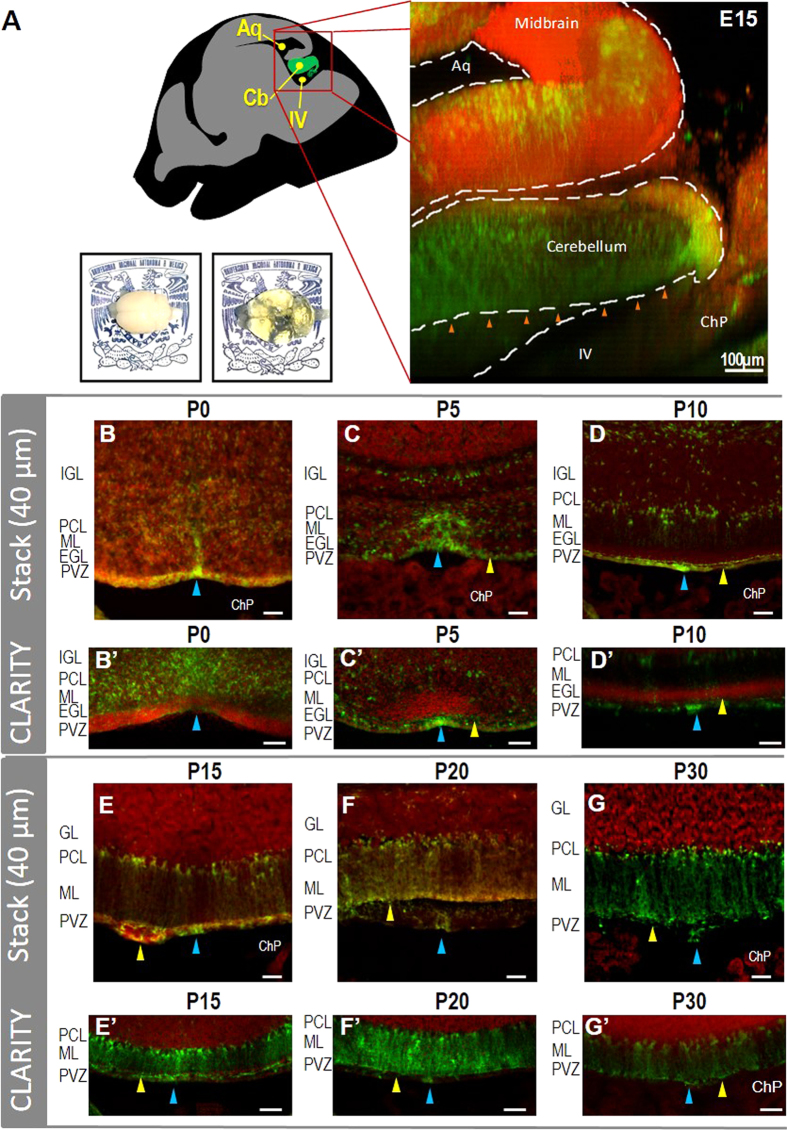
Development of the SVCC and VMC. The VMC is observed from embryonic (**E**) day 15 (orange arrowheads in **A**) in a 2-mm sagittal section of a clarified GFAP-GFP cerebellum (see also supplementary videos). From P0 to P5 the SVCC and VMC expand, whereas from P10 the VMC is ventromedially limited to the ependymal glial cell layer (blue arrowheads in B-G stacks of 40 μm and optical slices from CLARITY **B’–G**’). From P5 to P30 the SVCC was found to be delimitated by the end feet of the Bergmann glia (yellow arrowheads in 40-μm stacks [C-G] and optical slices from CLARITY [**C’–G’**]) of transgenic GFAP-GFP mice (red nuclei labeled with propidium iodide). An example of clarified adult brain is presented in the bottom in A. IGL: Internal granular layer; PCL: Purkinje cellular layer, ML: Molecular layer, EGL: external granular layer, GL: granular layer; PVZ: Periventricular zone, ChP: Choroid plexus, P: postnatal stage. Barr: 50 μm.

We analyzed the glial organization of the SVCC and VMC during pre- and postnatal development. We obtained consecutive coronal sections of cerebella from transgenic GFAP-GFP mice processed for CLARITY (Fig. 7). From 13 slices of lobe I at P0 we observed that the SVCC is formed by GFP^+^cells apparently in migration towards the white matter (Fig. 7B–G, blue and yellow arrowheads. This organization suggests that this section may be a niche of glial cells actively proliferating during early postnatal development, since after P5 the number of GFP^+^cells decreased, and the label was limited to the central section of the SVCC and the ependymal glial cell layer (blue arrowhead in Fig. 7C,C′), whereas in the adult (P30), the GFP^+^cells remained only in the VMC (blue arrowhead in Fig. 7G,G′).

Analysis of cerebella processed by CLARITY showed that in the SVCC numerous GFP-expressing cells are present from E15 and probably correspond to sites of gliogenic activity (orange arrowheads in Fig. 7A and supplementary Fig. 4). In the P0 cerebellum the cells that express GFP form the VMC and are densely concentrated in the central area of the SVCC (Fig. 7B′); consistent with observations in slices of cerebellum, the cells that express GFP were confined to the central region of the SVCC and form the VMC along the roof of the ventricle (blue arrowhead in Fig. 7B′–G′).

All these observations indicate that the SVCC expands during early development and in the adult, it remains as a niche that contains a variety of cells including astrocytes, oligodendrocytes and neurons.

## Discussion

**Figure 8:**
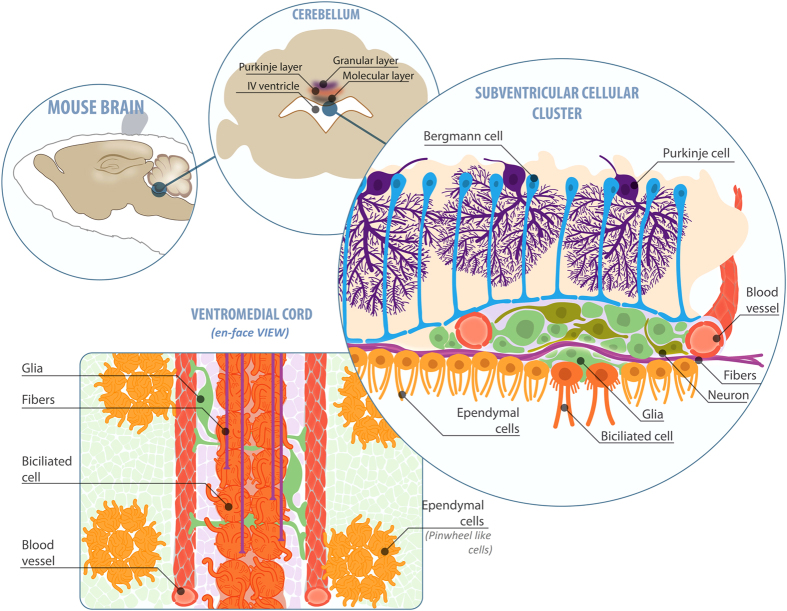
Organization of the SVCC and VMC. The drawing summarizes the organization of the peri- and subventricular zones in cerebellum. Top right, the SVCC is dorsally limited by the terminal feet of Bergmann glial cells (blue) and ventrally by multiciliated (yellow) and biciliated (orange) ependymal cells. Two capillaries are lateral to this structure (red). The glial cells (soft green) contact the blood vessels, as well as neurons (dark green) and myelinated axons (purple). Lower left, the VMC on the roof of the fourth ventricle is composed of two rows of biciliated cells (orange); fibers (purple) and glial cells (soft green) extend their processes towards two capillaries (red). The presence of pinwheel-like structures was also evidenced (yellow). Illustration by F. Serrano.

Some of the results considered in this report are based on previous observations that disclosed the presence of diverse electrophysiological profiles of cells distributed along the periventricular zone of cerebellum^1^. In brief, ependymal glial cells, astrocytes, oligodendrocytes and neurons were detected in lobules I and X that form the roof of the fourth ventricle. Figure 8 summarizes the findings reported in this paper. This zone of the cerebellum has not been widely studied, but the organization of ependymal cells and their responses to GABA have been documented^1^,^2^,^9^,^17^. In contrast, substantial evidence for a diversity of cellular phenotypes in periventricular zones comes from studies of the lateral ventricles^18^,^19^ and the central canal^20^,^21^; these cells are associated with neurogenic niches or homeostatic control.

In adult cerebellum the presence of niches with active proliferating cells have been documented in white matter and in the Purkinje cellular layer, giving rise to GABAergic interneurons and astrocytes, respectively^22^,^23^ but this level of specialization has not been observed in the periventricular zone of cerebellum. Thus our unexpected anatomical observations presented in Fig. 1 and the electrophysiological responses previously reported^1^ prompted us to determine the properties of this cluster of cells.

Lobules I and X of the cerebellum face the ventricle, and their organization is clearly specialized to communicate intimately with the CSF. Bergman glial cells of lobules II to IX send their processes radially, and their foot-plates contact the basal lamina and form the glia limitans; there is little or no interaction with astrocytes in this zone, which contrasts with the mainly astrocytic composition of the glia limitans in the forebrain and spinal cord^24^. Towards the central region of the ventricular face of the cerebellum, the end-feet of the Bergmann cells project to the dorsal side of the SVCC, where they contact blood vessels and surround neurons that project transversally. The SVCC includes diverse cellular components that we found to be positive for the following identity markers: a) nestin, b) GFAP, c) vimentin, d) MBP, e) NeuN, f) GAD65, and g) calretinin. All these markers confirm the presence of astrocytes, oligodendrocytes and neurons that were identified previously by electrophysiology^1^. The SVCC was not labeled by antibodies against NG2. The myelinated axons running transversally and longitudinally along the dorsal border of the SVCC and ependymal glia are of unknown origin (Fig. 2D), and it is yet to be determined whether these neurons form a local circuit within the cerebellum or come from other regions of the brain. On the other hand, the electrophysiological responses from glial cells shown in Fig. 6 correspond to astrocytes, although the morphological characteristics of these cells (small soma and long main processes running laterally accompanied of shorter processes), are not the typical characteristics of astrocytes from cerebellum^22^,^25^. This may suggest novel morphologies of the cell type in the SVCC with potential different functional characteristics.

The presence of fenestrated capillaries in the SVCC was disproven by the null penetration of Evans blue into the area, although extensive vasculature was found. Extravasation of Evans blue was observed in the choroid plexus and other circumventricular organs, indicating that the labeling was effective. The absence of fenestrated capillaries makes it improbable that the SVCC forms a structure with functions like those of CVOs^26^,^27^,^28^, since intimate communication of cells, either glia or neurosecretory neurons, with blood would be blocked by tight junctions preventing a functional neuroendocrine system. In addition, the limited labeling of BrdU and Ki67 in the SVCC suggest that, despite the presence of nestin^+/^GFAP^+^cells, extended blood irrigation and contact with the CSF, this area is not a neurogenic niche, such as that found in the lateral ventricles^16^, this was supported by the absence of cells expressing the immature neuronal marker DCX (not shown). Cells of the floor of the fourth ventricle can be induced to proliferate and differentiate into astrocytes and oligodendrocytes when mice are perfused with a mix of FGF2, EGF and heparin^29^ and it had been reported that in adult New Zealand white rabbit, neurogenesis take place in some restricted niches located in the subpial zone^30^. It is well documented that different cellular populations in the cerebellum, including inferior olivary neurons and Purkinje cells have more plasticity than previously thought^31^,^32^,^33^. We have not yet explored whether the cells from the SVCC have these abilities, but it is a possibility worth studying.

The ventricular position of the SVCC and VMC, their cellular organization and the considerable number of GFAP^+^cells that compose them during ontogenic development suggest that they may be an active niche of proliferative cells that is spatially restricted after cerebellar development is completed (Fig. 7). CLARITY and coronal slices of transgenic GFAP-GFP mice revealed that the SVCC and VMC occupy a larger proportion of the area during early development than in adulthood. A stream of GFAP^+^cells in apparent migration towards the parenchyma is observed in newborn mice; this organization is transformed in such a way that, after the second week of age, GFAP^+^cells from the VMC, are reduced to only a few cells in coronal slices, and are more apparent in the *en-face* preparation. The VMC forms an array that is structurally similar to the rostral migratory stream of the lateral ventricles; indeed, several disperse, pinwheel-like structures were detected (Fig. 3D and F), similar structures are associated with adult active neurogenic niches in the lateral ventricles^15^,^34^,^35^; however, as previously shown, no evidence of active cell proliferation was detected. Further studies will be necessary to determine the role of these organizational features in this area of the cerebellum.

On the surface of the central region of the roof of the fourth ventricle we found a cellular complex that forms a cord running along the medial line and is composed of nestin^+^and GFAP^+^cells. We have named this structure the Ventromedial Cord or VMC that, due to the morphology of GFAP^+^cells, appears to be composed of cells in migration (Fig. 3); however, despite applying several experimental strategies (not shown), we did not observe active movement of these cells. The VMC accompanies a series of blood vessels associated with GFAP^+^cells that contact the CSF (most probably astrocytes and/or pericytes); these cells are in a strategic position that permits sensing the conditions of the circulatory system and CSF. These cells possess microvillosities and two cilia unlike the ependymal glial cells that possess multiple cilia, are widely distributed on the roof of the ventricle and have the morphology previously observed of ependymal glial cells^2^.

The results shown here do not unequivocally demonstrate a functional role for the cluster of cells described; we do not know whether the cells from the VMC and SVCC transform into glia or neurons, or if they are migrating along the surface of the ventricle. High proliferative and differentiation potentials of the cells within these niches would present considerable discrepancies with the general view of limited neurogenic and gliogenic activity in the adult cerebellum. It is interesting, however, to speculate that they may form a novel specialized structure with an unknown function, and their association with novel myelinated axons makes the SVCC even more intriguing. It will be a fascinating challenge to develop a strategy to resolve this puzzle. For now, it is worth mentioning that the SVCC has been observed in three different species of rodents, in rat: *Ratus norvegicus,* in *Neotomodon alstoni* and in the North American prairie vole: *Microtus ochrogaster (not shown)* which suggests that it is evolutionarily preserved.

## Additional Information

**How to cite this article**: González-González M. A. *et al*. Identification of novel cellular clusters define a specialized area in the cerebellar periventricular zone. *Sci. Rep.* **7**, 40768; doi: 10.1038/srep40768 (2017).

**Publisher's note:** Springer Nature remains neutral with regard to jurisdictional claims in published maps and institutional affiliations.

## Supplementary Material

Supplementary Information

Supplementary Video 1

Supplementary Video 2
