# Supplementary material for: Identification of novel cellular clusters define a specialized area in the cerebellar periventricular zone
Source: Sci Rep. 2017 Jan 20;7:40768. doi: 10.1038/srep40768 (PMC5247769; doi:10.1038/srep40768)
Supplement: Supplementary Information [file srep40768-s1.pdf]

Identification of novel cellular clusters define a specialized area in the cerebellar periventricular zone

González-González María Alejandra[1], Gómez-González Gabriela Berenice[1], Becerra-González Marymar[1], Martínez-Torres Ataúlfo[1]*.

[1]Instituto de Neurobiología, Universidad Nacional Autónoma de México, Departamento de Neurobiología Celular y Molecular, Laboratorio de Neurobiología Molecular y Celular, Juriquilla, Querétaro, 76230, México.

Corresponding author: Dr. Ataulfo Martínez-Torres. ataulfo@unam.mx.

Supplementary information

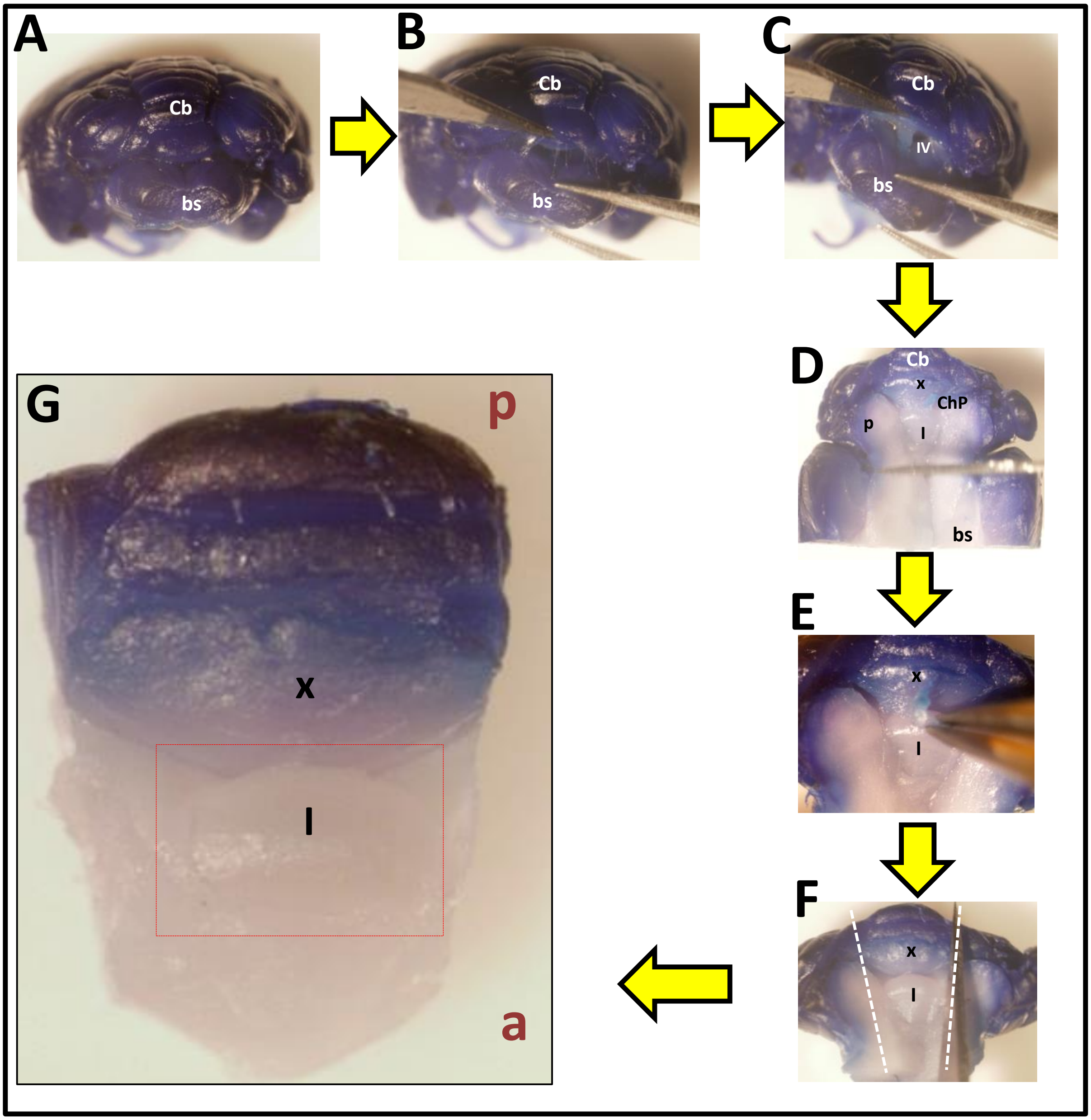

Supplementary 1. Whole-mount dissection of cerebellum. Whole cerebellum was stained with toluidine blue in order to contrast the roof of the ventricle (clear). A. Panoramic view of the brain includes cerebellum (Cb) and brain stem (bs). B and C. Mechanical separation of the Cb and bs permits observation of the fourth ventricle (IV). D. Using a dissection knife the cerebellar peduncles are removed, exposing the I and X lobules (I and X respectively), the ChP and the dorsal portion of the brainstem (bs). E. ChP and lateral sections of cerebellum are removed to show the surface of the I and X lobes (F). Final dissection is presented in G, in which a square indicates the area that corresponds to the zone of interest. a: anterior; p: posterior.

.

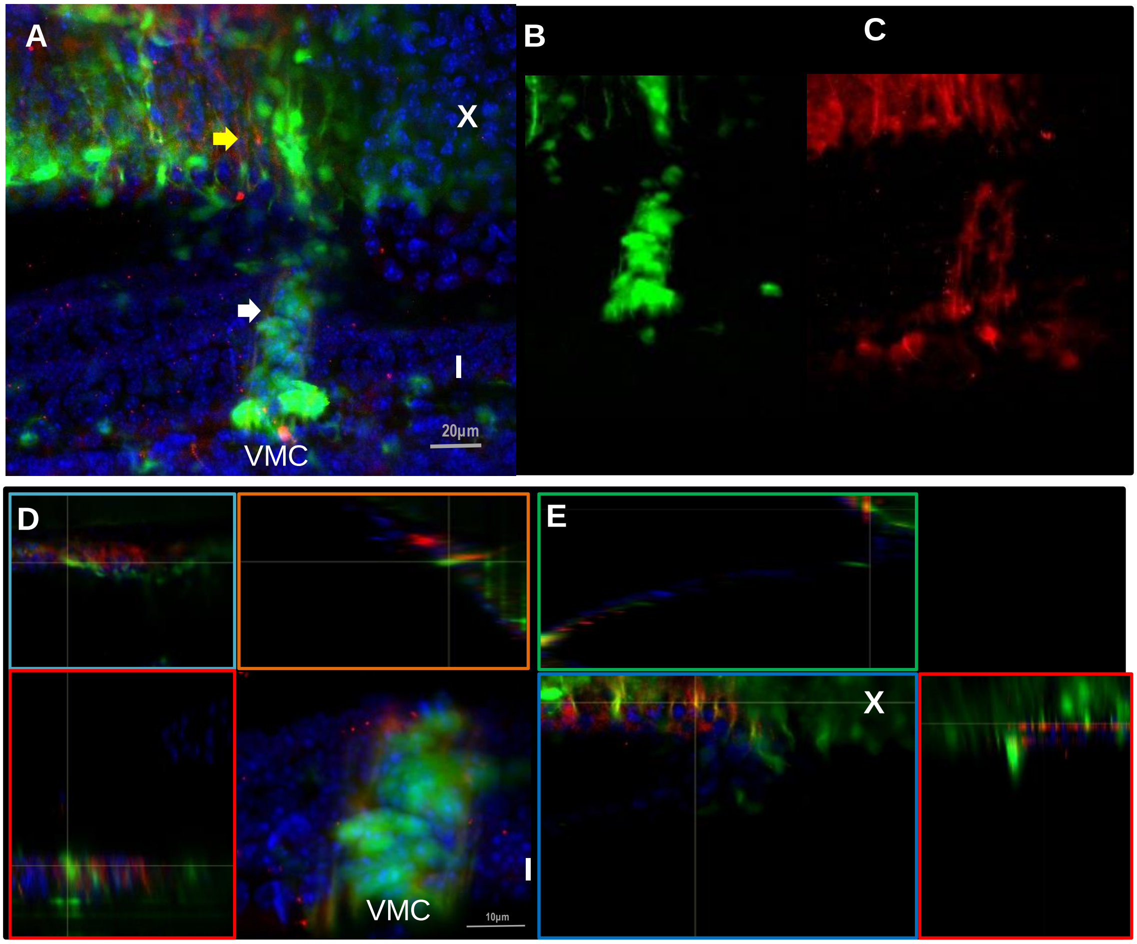

Supplementary 2. GFAP and nestin^{+} cell distribution in the VMC. Orthogonal projections of the GFAP^{+} and nestin^{+} cells of the SVC. A *en-face* view of the VMC. White and yellow arrows point to the area observed in D and E, respectively. B and C show the distribution of GFAP^{+} (green) and nestin^{+} (red) cells. D. Orthogonal view of cells expressing both markers. E. Organization of GFAP^{+} nestin^{+} cells. I, lobe 1; X, lobe 10, VMC, ventromedial cord.

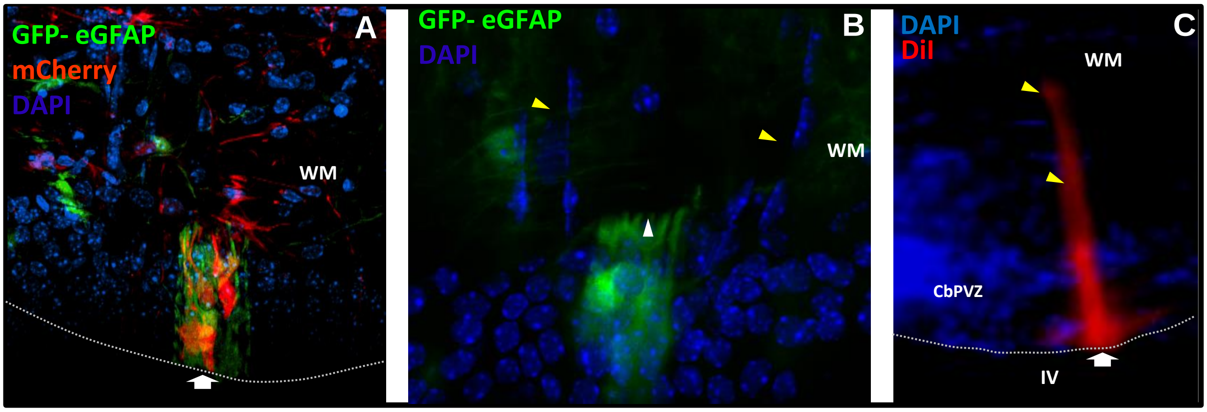

Supplementary 3. Organization of the VMC in coronal section. A. mCherry labeling (cells in red) accompanied by glial cells (green) disclosed that cells of the VMC extend projections to the parenchyma and directly to the white matter (wm), which is also shown. In B, the projection ends near two blood vessels (yellow arrowheads). DiI labeling also shows the VMC (arrow) projecting to the parenchyma and towards the wm (yellow arrowheads in C).

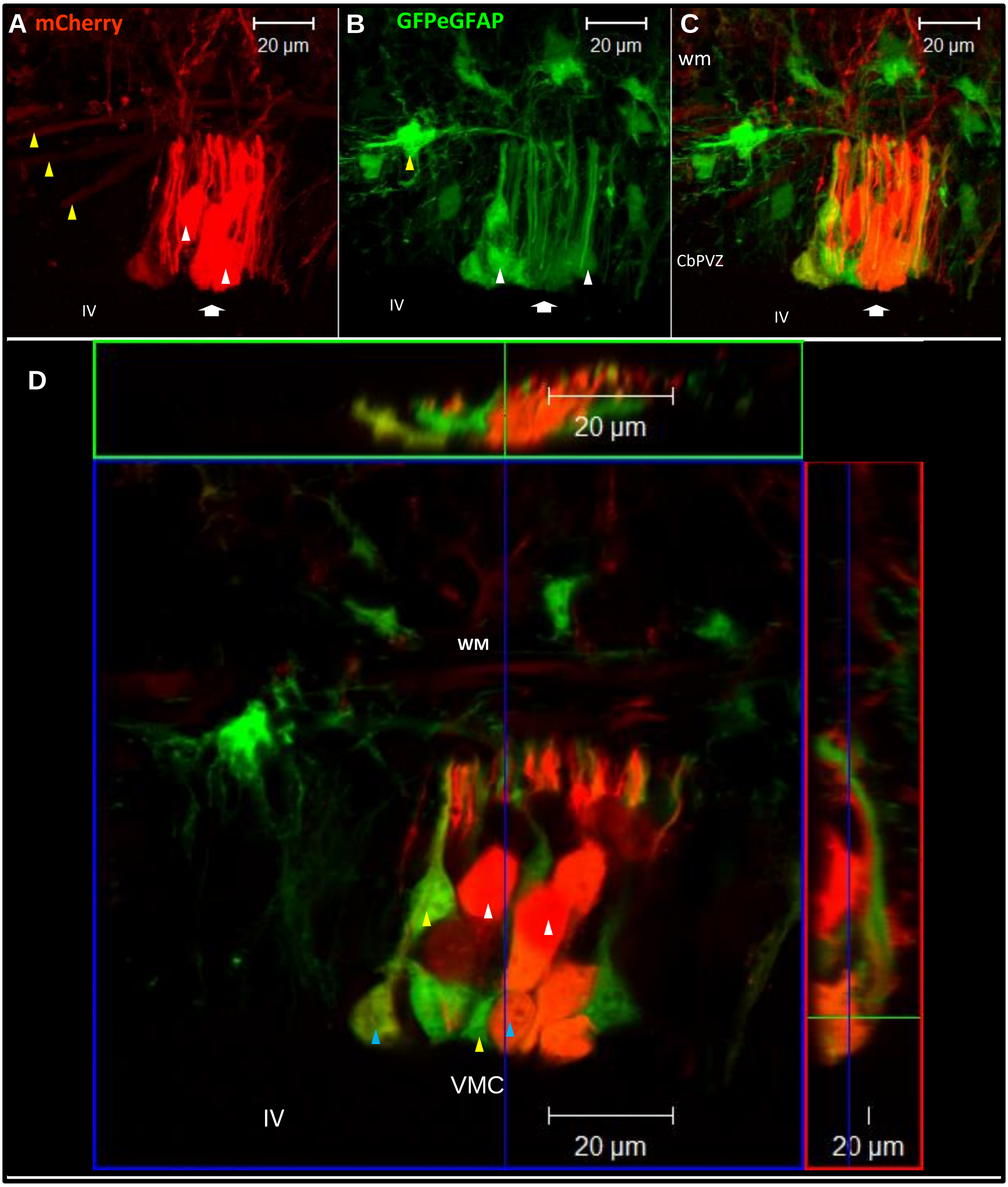

Supplementary 4. Organization of the VMC includes GFAP^{+} and GFAP^{-} cells. The VMC in coronal sections (arrow). mCherry label (white arrowheads in A) intermingled with GFP-labeled cells; projections of cells around the area are indicated by yellow arrowheads. Merged images are shown in C. In D, an orthogonal projection revealed that the VMC includes GFAP^{+} and GFAP^{-} cells, and an astrocyte (shown in B with a yellow arrowhead) extends its projections to the VMC. WM, white matter.

Supplementary 5. Adenoviral tracing of the VMC (animation).

Supplementary 6. Glial cell distribution in the SVCC and VMC at E15 (CLARITY animation).
